# A comparison of Myribase and Doublebase gel: Does qualitative similarity of emollient products imply their direct interchangeability in everyday practice?

**DOI:** 10.1111/dth.14020

**Published:** 2020-07-29

**Authors:** Milica Lukic, Ivana Pantelic, Snezana Savic

**Affiliations:** ^1^ Faculty of Pharmacy, Department of Pharmaceutical Technology and Cosmetology University of Belgrade Belgrade Serbia

**Keywords:** dry skin, emollients, similar composition

## Abstract

Emollients are acknowledged as a part of standard care in therapeutic and prevention protocols as well as a part of everyday skin care routine. When it comes to making a final decision between two emollient products, the ingredient list, that is, the formulation composition could be the determining factor. In such cases the consumer, and some healthcare providers, believe that products with the same qualitative composition (ingredient list) must have the same efficacy. In this study, we have investigated the skin hydration performance of two emollient preparations (DBG and MBG), which appear to contain the same ingredients, and hence, could be considered interchangeable in everyday practice. Our studies showed that the effects of DBG were overall superior to the ones attributed to MBG at each investigated time point (1, 2, 4, and 24 h post application) when tested on normal and dry skin. Consequently, it is shown that two apparently qualitatively identical products do not necessarily provide matching efficacy.

## 1 INTRODUCTION

In topical products, the formulation itself and the specific microstructure, which is the result of complex interplay of formulation compounds, is recognized as one of the products critical quality attributes.^1^, ^2^, ^3^, ^4^ For emollients, which lack in conventional therapeutically “active” substances, efficacy depends on the performance of the whole formulation.

The use of emollients in everyday life is extensive and their importance is well recognized for both medical and cosmetic purposes.^5^, ^6^, ^7^ Emollients are acknowledged as a part of certain therapeutic protocols such as atopic dermatitis and various dry skin conditions.^6^, ^8^, ^9^, ^10^ Nevertheless, there are no specific requirements and/or recommendations regarding vehicle systems and types of moisturizers used in dermatological therapy.^11^, ^12^

For the prescribed emollients, it is a physician who commonly defines its use and their decision making is usually supported by the best‐practice statements and guidelines.^13^, ^14^ On the other hand, for an average consumer of cosmetic products, decision making is based on a range of different attributes and marketing information. However, when it comes to making a final decision between two emollient products, the ingredient list, that is, the formulation composition is likely to have a major impact. In such cases not only that consumers/patients but also clinicians/policy makers believe that the products with the same ingredient list have the same efficacy.

In the light of emollient importance in dry skin condition treatment, the majority of studies involving emollients address dry skin, but the normal skin, which can also experience occasional episodes of dryness, deserves the same consideration.^15^, ^16^ Also, studies concerning efficacy of emollients are mainly focused on the efficacy of an “active” in emollient products or on the correlation between different types of vehicles, but there is a lack of relevant, published data addressing both well‐established emollients and new emollient formulations.^17^, ^18^, ^19^, ^20^, ^21^

Here, we highlight important efficacy shortcomings of the assumption that two quantitatively the same emollient products can be used therapeutically interchangeable by presenting the results of our studies. It was our aim to compare the human skin moisturisation effects of two qualitatively the same emollients within different products categories prescribed by UK doctors. As representative products which satisfied this aim, Doublebase Gel (Diomed Developments Ltd—a licensed medicine) and Myribase Gel (Penlan Healthcare—a Class I medical device and branded generic), were investigated. To the best of our knowledge, similar studies that may be relevant to everyday physicians' practice have not been published.

## 2 MATERIALS AND METHODS

The investigated preparations MyriBase Gel (MBG) and Doublebase Gel (DBG) both list the following ingredients: Water, Isopropyl Myristate, Liquid Paraffin, Glycerol, Carbomer, Sorbitan Laurate, Triethanolamine, and Phenoxyethanol. The qualitative composition was provided on the packaging material, but information related to quality standards of ingredients, the quantitative composition, or to the manufacturing process, were not disclosed. However, both products are labeled to contain 15% of each of Isopropyl Myristate and Liquid Paraffin.

In order to investigate the emollients' efficacy, 40 volunteers (both genders with normal, healthy skin) participated in each study.

The studies were conducted on volar aspects of both forearms (randomization established in advance) as double‐blind, randomized, and bilateral. Two test sites (25 cm^2^ each) were used for the application of investigated (MBG and DBG) products, while the third site served as a non‐treated control. The study was conducted in compliance with the Declaration of Helsinki and in accordance with Good Clinical Practice, valid guidelines and recommendations, and approved by the local Ethics Committee (approval No. 2458/2).^22^

The volunteers were obliged not to use other products on the test sites 2 days before the study as well as throughout the study. During the study they were not permitted to bath or shower. Prior to each measurement, the volunteers rested in acclimatized premises (*t* = 22 ± 1°C and RH = 35 ± 5%) for 30 min. The stratum corneum moisturization (SCM) level measurements were performed by Cutometer MPA 580 with an integrated CorneometerCM 825 (Courage & Khazaka, Germany).

### 2.1 Study 1 protocol

The levels of SCM were measured before product application to obtain the initial/baseline values. Subsequently, 4.5 μL/cm^2^ of the samples were applied by the investigator to the defined test sites. The third test site served as the non‐treated control. After sample application, corneometry readings were conducted after 1, 2, 4, and 24 h. The readings for each test site were expressed as the mean values of 15 measurements.

### 2.2 Study 2 protocol

In order to induce dryness in otherwise healthy skin, a previously established protocol was used in accordance with relevant guidelines.^23^ Sodium lauryl sulphate (SLS), 10% (w/w) solution in 100 μL, was applied to test sites (25 cm^2^). On the top of a cotton pad impregnated with the SLS solution, an occlusive film (Parafilm, Germany) and a hypoallergenic adhesive tape (Sensifix, Serbia) were placed. The entire SLS patch was removed after 6 h and the treated skin was rinsed with tap water. A baseline measurement was taken 48 h after the dry skin induction. After successful dry skin induction, the study protocol was equivalent to the one described in Study 1.

### 2.3 Statistical analysis

Statistically significant changes were quantified at two levels: five different time points (0 [basal/initial value], 1, 2, 4, and 24 h post sample application) for each treatment, and three different treatments (DBG application, MBG application and non‐treated control NC—no application). In order to investigate the difference, two‐way ANOVA was performed and a post‐hoc *Bonferroni t‐test* was conducted where appropriate, for all pairwise comparisons within the data set.

Statistical analysis was carried out using SigmaStat (Version 3.1, Virginia) with significance levels set at *P* < .05.

## 3 RESULTS

**FIGURE 1:**
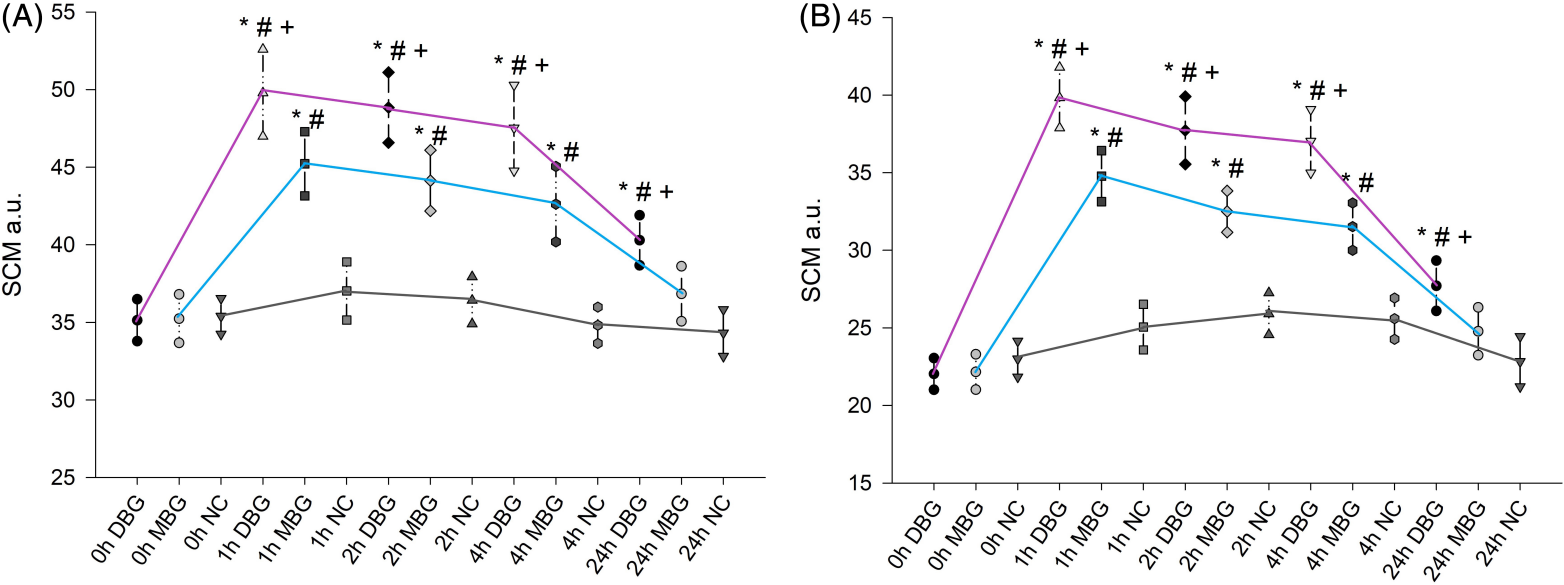
A, Stratum corneum moisturization (SCM) mean values (a.u) with 95% confidence intervals obtained in the Study 1 (normal skin) and B, Stratum corneum moisturization (SCM) mean values (a.u) with 95% confidence intervals obtained in the Study 2 (dry skin); violet line—DBG treatment, blue line—MBG treatment, and gray line—non‐treated control (NC); statistically significant difference compared to *—basal values (*P* < .001), #—non‐treated control (*P* < .001) and +—another treatment (*P* < .05)

In Figure 1A, results obtained on normal skin (Study 1) are presented. After application of DBG, SCM values were significantly increased after 1, 2, 4, and 24 h, when compared to baseline values and non‐treated control. Changes between SCM values at different time points and baseline were after 1 h: 14.7 ± 6.6 (*P* < .001), 2 h: 13.7 ± 5.9 (*P* < .001), 4 h: 12.4 ± 6.8 (*P* < .001), and 24 h: 5.2 ± 4.6 (*P* < .001). Differences obtained within the same time point for non‐treated and DBG‐treated skin were at 1 h: 12.8 ± 8.7 (*P* < .001), 2 h: 12.4 ± 6.4 (*P* < .001), 4 h: 12.6 ± 6.7 (*P* < .001) and 24 h: 6.0 ± 5.0 (*P* < .001). There were no significant differences between the results obtained after 1 and 2 h, nor between the results obtained after 2 and 4 h. After application of MBG, SCM values were significantly increased after 1, 2, and 4 h, when compared to the respective baseline values and the non‐treated control, somewhat similar to those attained for DBG. For MBG treated skin mean SCM values increase after 1 h was 10.0 ± 4.9 (*P* < .001), 2 h: 8.9 ± 5.4 (*P* < .001) and 4 h 7.4 ± 5.8 (*P* < .001), and compared to non‐treated area at 1 h it was 8.2 ± 7.0 (*P* < .001), 2 h: 7.7 ± 6.6 (*P* < .001) and 4 h: 7.8 ± 6.8 (*P* < .001). However, 24 h after the application of MBG the increase was not statistically significant (SCM values have been changed compared to baseline and NC for 1.6 ± 4.9 and 2.5 ± 4.7, respectively).

Since the two investigated products appear to have the same qualitative composition, it was of interest to investigate whether their efficacy is also the same. In Figure 1A, it could be observed that SCM values were more increased after DBG compared to MBG application at every time point. The fact that the DBG sample induced a significant increase after 24 h in skin moisturization levels while the MBG sample did not, indicates differences in their efficacy.

Study 2 was conducted on chemically induced dry skin and obtained results are presented in Figure 1B. After application of DBG, the results attained for dry skin were comparable to those for normal skin. Skin moisturization was significantly increased after 1, 2, 4, and 24 h, when compared to baseline values (differences after 1 h: 17.8 ± 4.6, 2 h: 15.7 ± 6.3, 4 h: 15.0 ± 5.1, 24 h: 5.7 ± 4.9; *P* < .001) and to the non‐treated control (differences at 1 h: 14.8 ± 5.8, 2 h: 11.8 ± 6.9, 4 h: 11.4 ± 7.4, 24 h: 4.9 ± 4.1; *P* < .001).

Following MBG application to the dry skin, findings were again comparable to the Study 1. Therefore, when applied onto dry skin, MBG initially increases the moisture content, up to 4 h, but after 24 h the increase could not be considered statistically significant.

When the results of these different treatments are compared, DBG moistutization effect was significantly better when compared to the effect of MBG in every assessed time point. SCM values were significantly higher after DBG treatment for 5.0 ± 5.2 (*P* < .05) after 1 h, 5.2 ± 5.7 (*P* < .05) after 2 h, 5.5 ± 6.1 (*P* < .05) after 4 h and after 24 h for 2.9 ± 3.8 (*P* < .05).

## 4 DISCUSSION

The results indicate that both emollients provide significant moisturization effects 4 h after the application on normal, as well as on dry skin. The fact that investigated MBG did not significantly increase SCM levels of either normal or dry skin 24 h after the application, implies the necessity of more frequent MBG application in order to obtain a satisfactory emollient effect. However, it is generally accepted that many patients fail to adhere to the recommended frequent emollient application.^24^

When the effect on normal and dry skin between samples is compared, the DBG moisturization effect was better when compared to the effect of MBG at every assessed time point after the application. The relevance of these findings suggests that, regardless of whether the compared emollient formulations appear to be qualitatively the same, their inherent microstructure may have a profound effect on the product performance.

For licensed medicines, regulatory authorities require generic products to be both pharmaceutically equivalent and bioequivalent to the innovator in order to substantiate therapeutic equivalence and interchangeability. To establish bioequivalence, for most topically applied dermatological products, comparative clinical endpoint trials are required. For the post approval changes, in order to reassure regulators that changes have not compromised bioequivalence, manufacturers have to follow regulatory guidance, which are not restricted to clinical trials but in vitro methods could be used as well.^25^ For generic substance‐based Class I medical devices registered in the EU, little or no attention is given to bioequivalence. The results of the studies presented here demonstrate that the skin moisturization effects of MBG are inferior to the innovator product, DBG, in relation to both the magnitude and duration of the hydration effect. Although Class I medical devices contain no active ingredients as such, their qualitative and quantitative compositions as well as their production methods, equipment, etc. are equally important and more attention should be given to their investigations.

Taking into account that both emollient preparations appear to have similar compositions, and the fact that quantitative formulation and employed manufacturing processes were unknown to the investigators, the observed difference in skin moisturization performance may be attributed to the difference in the two emulsion systems' microstructure. This is in line with the three levels of product equivalence commonly controlled by the regulatory agencies: qualitative (Q1) and quantitative (Q2) composition and the arrangement of matter and microstructure of topical formulations.^3^ Namely, according to the composition declared by both manufacturers, their similarity is evident at the Q1 level, but the Q2 level equivalence cannot be assumed.

The quantitative aspect of particular ingredients, alongside the selection of specific manufacturing parameters, determine the final microstructure and sensory properties of an emulsion type product.^26^, ^27^ Within the investigated products, stabilized via the combination of a typical hydrophilic polymer (Carbomer) and a predominantly lipophilic surfactant (Sorbitan laurate), a unique emulsion system is formed, offering the possibility for diverse modes of water immobilization and plenty of variations in textural and rheological behavior. This scope for diversion is further complicated by the absence of data standardizing the quality of each ingredient. The names of the ingredients may be identical, but that provides no guarantee of interchangeability as regards chemical and physical conformity.

Obtained results are useful and relevant within the scope of our investigation, but it is important to mention that they may not necessarily apply to people with certain dry skin conditions, and achieved differences in SCM may not be translated into clinically important for some skin disorders, since our studies comprised healthy participants.

## 5 CONCLUSION

The obtained results are directly applicable in an everyday physician's practice, since they confirm that a simple choice of apparently the same ingredients/formulation components is not sufficient for complete interchangeability of formulations. Our study contributes to the standpoint that decisions on the appropriate emollient treatment should be founded on evidence‐based information on actual product efficacy, since other critical quality attributes, such as quality of the material used, the inherent microstructure and the general arrangement of matter within the formulation, may prove to have a decisive influence on the treatment outcome.

The investigated emollient preparations successfully elevated skin moisturization in normal and induced dry skin 4 h after single application. Yet only the DBG provided statistically significant SCM increase 24 h after the treatment, implying its capability to provide prolonged skin moisturization. Additionally, comparative analysis of the two treatments revealed that all SCM values attributed to the DBG treatment were significantly elevated at each assessed time point, relative to the MBG treatment.

## CONFLICT OF INTEREST

The authors declare no conflict of interest.
